# Consumers’ Implicit and Explicit Recall, Understanding and Perceptions of Products with Nutrition-Related Messages: An Online Survey

**DOI:** 10.3390/ijerph17218213

**Published:** 2020-11-06

**Authors:** Beatriz Franco-Arellano, Lana Vanderlee, Mavra Ahmed, Angela Oh, Mary R. L’Abbé

**Affiliations:** 1 Department of Nutritional Sciences, Faculty of Medicine, University of Toronto, Medical Sciences Building, 1 King’s College Circle Rm 5368, Toronto, ON M5S 1A8, Canada; beatriz.francoarellano@mail.utoronto.ca (B.F.-A.); lana.vanderlee@fsaa.ulaval.ca (L.V.); mavz.ahmed@utoronto.ca (M.A.); angela.oh@uottawa.ca (A.O.); 2 École de Nutrition, Université L and Al, Pavillon des Services, Bureau 2729-E, 2440 Boul. Hochelaga, Quebec City, QC G1V 0A6, Canada; 3 Faculty of Law, University of Ottawa, 57 Louis Pasteur St, Ottawa, ON K1N 6N5, Canada

**Keywords:** nutrition labelling, nutrition claims, symbols, consumer perceptions, nutrient declarations, Canada

## Abstract

This study aimed to assess consumers’ implicit and explicit recall, understanding and perceptions of products with a nutrition claim and a symbol depicting ‘health,’ and to determine whether these perceptions differed among Nutrition Facts table (NFt) users vs. nonusers. In an online survey, participants (n = 1997) were randomized to one of eight conditions in a 2 × 2 × 2 factorial design, consisting of a label with a claim (present/absent) a heart-shaped symbol depicting ‘health’ (present/absent) for a healthier or less healthy soup. Participants were shown a label for 10 s and asked whether they recalled seeing a claim. If participants answered yes, they were then asked to describe their response using open-ended questions. Participants also rated the product’s perceived nutritional quality and purchase intentions using seven-point Likert scales. In the claim condition, most participants (75%) were able to recall the presence of a claim, while 12% incorrectly mentioned the presence of a claim when there was none. Claims likely attracted consumers’ attention and increased perceived nutritional quality, although with limited influence among NFt users (23%). The symbol depicting ‘health’ did not enhance perceived nutritional quality or purchase intentions. Although most participants (77%) made their decisions implicitly using the front of labels, those who used the NFt had a better understanding of the nutritional quality of products.

## 1. Introduction

In past decades, psychologists and consumer behavior scientists have investigated consumers’ evaluation of products and decision-making [1]. Psychologists have classified how people make decisions in two ways: One that is fast, automatic, and effortless, which relies on preconceived beliefs, intentions, patterns, perceptions, intuition, and/or memory, commonly known as “System 1” [1]; and a second, which is slow, effortful, and conscious, which relies on cognitive reflection of options when decisions are being made, often called “System 2” [1]. Thus, it is not surprising that people use the former system more often than the latter to make decisions. 

When consumers retrieve information automatically and without reasoning (i.e., using System 1), this process is called an “implicit” evaluation [2,3]. An “explicit” evaluation occurs when consumers judge products through a thorough assessment of the available information on the product (i.e., using System 2) [2,4]. Implicit evaluations can be measured with response time-based methods and open-ended questions, while explicit evaluations can be measured through direct questioning using self-report methods, such as Likert scales [5].

There are many factors affecting food choices, such as the impact of brand and sponsorship [6,7,8], price [9,10,11,12], and health motivation [13,14,15]. However, time, nutrition education and knowledge, familiarity with foods, and visual attention to labels have been found to be among the most important aspects of decision-making during grocery shopping [9,16,17,18,19,20,21,22,23,24]. These factors can greatly determine which path (automatic/fast vs. conscious/slow) consumers may use to evaluate and purchase products [1]. For example, when consumers have less time to make a food choice, only certain product characteristics (e.g., price, content or absence of a particular nutrient, expiration date) might be evaluated by consumers before purchase [25,26]. Nutrition knowledge can also influence how information on a food label is processed by consumers [27]. Moreover, greater nutrition knowledge has been associated with label use [20,28]. Attention to labels can also predict food choice [17,18]. The more time consumers spend viewing a label, the more likely it is that product will be purchased [16,17,29,30]. Therefore, visual attention becomes critical for product selection when consumers are challenged with many options at supermarkets [16]. 

In recent years, consumers’ attention and interest in the health aspects of food products have risen [24,30,31,32,33,34,35,36]. Health-related messages, such as nutrition claims, images, and symbols, are frequently used on food labels, likely due to their ability to attract consumers’ attention [29]. Nutrition claims, which include nutrient content claims and health claims, are a voluntary component of nutrition labelling [37]. They can be found on a considerable proportion on food labels [38,39,40,41]. Whereas these claims are largely regulated by governments, some products that display nutrition claims can be of poor nutritional quality [39,42,43]. In Canada, regulations for nutrition labelling are outlined in the Food and Drug Regulations, which are governed by Health Canada [44]. Some nutrition labelling features, such as the Nutrition Facts table, Ingredients List, and other information (e.g., weight, allergen statements), are mandatory [44]. Although nutrition claims are a voluntary component of nutrition labelling in Canada, there are defined criteria that must be met if nutrition claims are displayed on food labels [44,45,46]. Moreover, nutrition claims not only can create a ‘halo’ effect on products and increase perceived product nutritional quality among consumers [47,48,49], but they also are chosen more often compared to products without claims [50]. In addition, qualitative research has shown consumers may not be able to distinguish between different claims types (i.e., nutrient content claims vs. health claims) [51,52]. 

Health-oriented images and symbols are also used to infer health-related benefits on products, which are known to alter people’s beliefs and perceptions [47,48,53,54,55,56,57,58]. Health-oriented images can increase trust [53,55], create a ‘positive’ attitude toward the general qualities of a product [56], be used to indicate a ‘natural’ product [57], or can be falsely used by consumers to recall nonexistent product attributes [58]. For example, an earlier Canadian study found that, when foods were presented with unregulated “positive” front-of-pack (FOP) symbols, products were perceived as healthier [47]. Another study found that even simple symbols, such as an image of a plant leaf, may lead consumers to overrate a product’s “healthiness” [48]. A recent study also found that health-related images increase perceived benefits and decreased perceived risk of consuming dietary supplements [56]. In contrast, nutrient declarations (called the Nutrition Facts table (NFt) in Canada), which are part of the mandatory nutrition labelling requirement in many countries, are often overshadowed by nutrition claims and health-related symbols due their position on the back of labels and overwhelming numerical content [59]. 

In Canada, nutrition claims are often displayed on food labels [60]. Yet, few studies have assessed how simple cues, such as health-related symbols rather than text, perhaps aided by intuition or prior nutrition knowledge, impact consumers’ understanding of claims. Thus, the primary objective of this study was to assess consumers’ implicit and explicit recall, understanding and perceptions of products, with a nutrition claim and a symbol depicting ‘health.’ It was hypothesized that labels with a claim and/or a symbol depicting ‘health’ would be perceived as healthier than comparable labels without a claim or symbol. As label use has been associated with greater nutrition knowledge [20,28], secondary objectives assessed whether consumers’ perceptions of products with claims differed among those who used the NFt (i.e., those who likely used an explicit evaluation) compared to nonusers (i.e., those who likely used an implicit evaluation), as well as the characteristics of NFt users. We hypothesized that most participants would not use the NFt. 

## 2. Materials and Methods 

### 2.1. Survey Design, Participants, and Initial Randomization

This study was a randomized controlled trial and part of a larger online consumer survey, which had individual tasks and research questions [61,62,63]. This study was registered at Clinicaltrials.gov (#NCT03290118), and the Health Sciences Research Ethics Board at the University of Toronto (Protocol ID#34393) reviewed and approved the study prior to it being conducted between September–October 2017, as described elsewhere [61,62,63]. Anonymized data were provided to the research team. Briefly, recruitment was conducted via email by a marketing company. The eligibility criteria consisted of age ≥18 years, English as primary spoken language, residency in Canada (territories excluded), some responsibility for household grocery shopping, ownership of a smartphone version iPhone 3 or later or Android, and the ability to able to complete the survey on a device with a minimum screen size of 9.7 in. The sampling criteria were established to be nationally representative as much as possible in terms of gender, age, and location, based on 2011 census data. However, other inclusion criteria (e.g., shopping habits, smartphone ownership) resulted in a final sample that was no longer representative (i.e., a greater proportion of participants had college/university education and were two years younger than the median Canadian population).

A link to the consent form and survey was emailed to each participant, who viewed and provided informed consent prior to the start of the survey. Participants were compensated with $10 or the equivalent in Air Miles^®^ if the survey was completed. Participants’ self-reported sociodemographic information (e.g., gender, age, education, income, ethnicity) was also collected. As part of the baseline data collection, participants answered a Canadian-adapted health literacy test (Newest Vital Sign) [64,65], in which participants were asked to interpret textual and numerical information from an NFt [64]. Participants were initially randomized to be in one front-of-pack (FOP) labelling condition: Control (no front-of-pack), warning labels (WL), health star rating (HSR), or traffic light labels (TLL). After randomization, participants were asked to test the FoodFlip©, which is a smartphone application that provides nutrition information using different front-of-pack symbols (WL, HSR, or TLL, and a control with an NFt). The results of the FoodFlip© trial have been described elsewhere [63]. For the purpose of this phase of the survey, no front-of-pack labelling was presented on any label, as it was not part of the objectives. The experimental design and stimuli for this particular study are described in the following section. The CONSORT diagram and checklist are provided in Supplementary Figure S1 and Table S1.

### 2.2. Experimental Design and Stimuli

**Figure 1:**
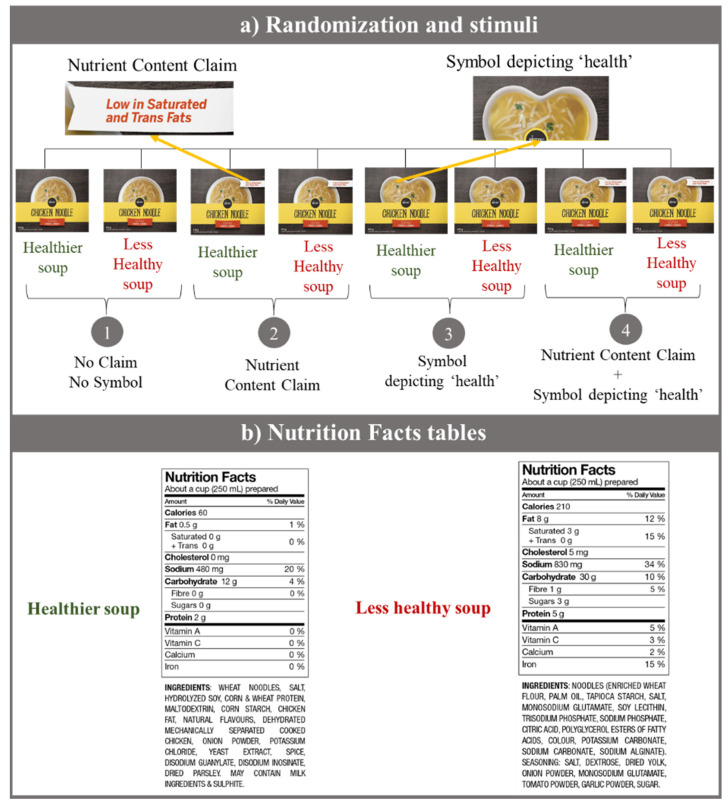
Images of mock labels used in the survey. (**a**) Summary of the eight conditions (2 × 2 × 2 factorial design) used in the survey and the four different labels created. (**b**) Nutrition Facts tables (NFts) for the healthier and less healthy soups. The nutritional quality was determined using the Food Standards Australia New Zealand Nutrient Profiling Scoring Criterion [67] and based on similar products found in the Canadian food supply.

Participants were re-randomized in a 1:1 ratio to one of eight conditions consisting of a label with a nutrient content claim (present, absent) and a symbol representing ‘health’ (present, absent) for soups of two different levels of nutritional quality (healthier, less healthy) (Figure 1a), in a 2 × 2 × 2 factorial design (8 groups, n = 250/group). The sample size was estimated to detect an effect size of 0.5 in the Likert scale, with at least 239 participants per group (power = 0.80, two-sided α = 0.05, and SD = 1.95). Randomization was conducted by the marketing company using an online computerized system. Mock labels of chicken noodle soups (same brand and design with four different variations) were created based on comparable products found in the Canadian packaged food supply [66]. A “low in saturated and trans fats” was selected as the nutrient content claim, since this type of product could qualify for such a claim and it can also be found on many similar products in grocery stores in Canada [60]. We also avoided a sodium-related claim, which is a claim often used on soups [60]. A heart-shaped bowl was selected as the symbol depicting ‘health,’ since images implying health functions are often (incorrectly) linked to health-related claims [58] and the heart symbol was identified as one used on Canadian food labels. The nutritional quality of soups was determined using the Food Standards Australia New Zealand Nutrient Profiling Scoring Criterion (FSANZ-NPSC), which is a nutrient profiling (NP) model used to determine the eligibility of products to carry health claims [67]. Briefly, in this NP model, a product gains ‘baseline points’ for its content of nutrients to limit (calories, sodium, saturated fat, and sugars), and points are deducted if a food contains nutrients or ingredients to encourage (protein, fiber, and fruits, vegetables, nuts and legumes) [67]. A final score is calculated and assessed against established cutoffs that, if exceeded, categorizes products as “less healthy.” Nutrition information for the “healthier” and “less healthy” soups are presented in Figure 1b. 

The experimental design was structured into two tasks, modelled after a method used earlier to detect implicit evaluations over nutrition and health claims, proposed by Klepacz and colleagues [58], and methods used to detect explicit evaluations by Wills et al. [24] and Wong et al. [53,68].

**Figure 2:**
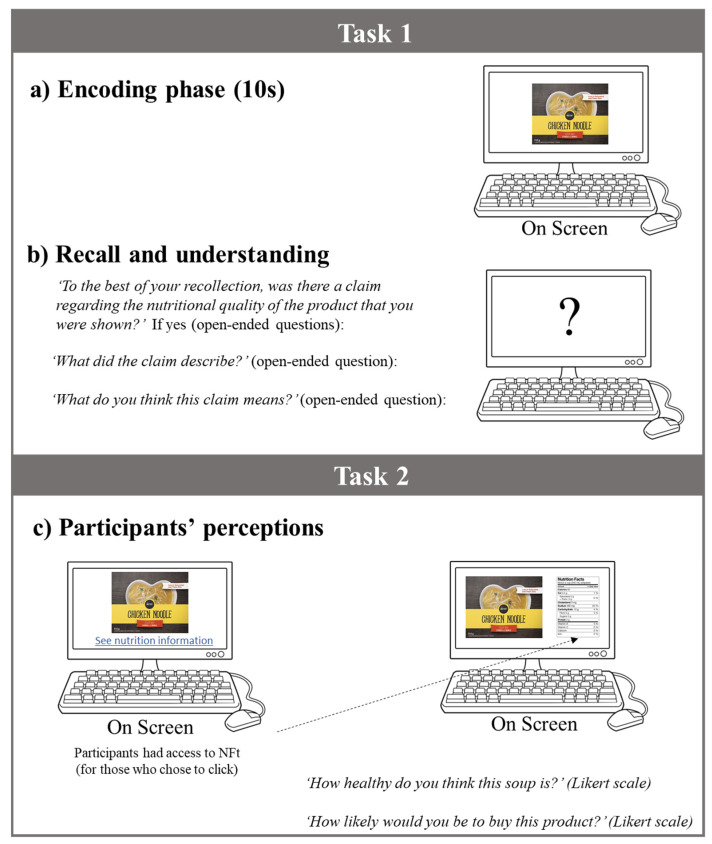
Study design and questions used in the survey. (**a**) Encoding phase: Participants were shown a label for 10 s. (**b**) Recall and understanding: Once the label was removed from the screen, participants were asked, ‘To the best of your recollection, was there a claim regarding the nutritional quality of the product that you were shown?’ If yes (open-ended questions): ‘What did the claim describe?’; ‘What do you think this claim means?’ (**c**) Perceptions: Participants were shown the same label again, which was left on the screen, and asked to rate product’s nutritional quality (‘How healthy do you think this soup is?’) and purchase intentions (‘How likely would you be to buy this product?’) using a seven-point Likert scale, where 1 was lowest nutritional quality/purchase intention and 7 was the highest nutritional quality/purchase intention. Participants had the option to click on the Nutrition Facts table.

Task one. This task had two phases: An encoding phase and a recall phase. In the encoding phase [58], participants were shown a label on the screen for 10 s (Figure 2a). In the recall or recognition phase [58], the label was removed from the screen, and participants were asked if they recalled seeing a claim on the label. Participants were also asked to describe their understanding of the claim using open-ended questions (Figure 2b). 

Task two. Participants were shown the same label again, which was left on the screen, and were asked to rate product’s nutritional quality and purchase intentions using seven-point Likert scales, where 1 was lowest nutritional quality/purchase intention and 7 was the highest nutritional quality/purchase intention. Participants had the option to look at the NFt by clicking at the hyperlink provided at the bottom of the screen, with no time restriction (Figure 2c). 

### 2.3. Statistical Analyses

Descriptive statistics and Kruskal–Wallis tests examined proportions of participants who recalled seeing a claim on the label and differences in those proportions, both overall and by condition. Responses to the open-ended questions were analyzed to identify key themes, which were later coded thematically and quantified. Generalized linear models with Bonferroni correction (adjusted for gender, education, income, ethnicity, and health literacy) were conducted to assess perceived nutritional quality and purchase intentions by condition and stratified by NFt use. We also conducted sensitivity analyses to determine whether results differed by gender and healthy literacy. 

## 3. Results

Participants’ characteristics (n = 1997) are presented in Table 1. Participants were between 18 and 85 years old, with an average age of 39 years. Of the respondents, 52% were female, half had university education, were mostly white, and had adequate health literacy. The initial results showed that the nutritional quality of the soups did not have significant effects on consumers’ recall (*p* = 0.63, as per the Kruskal–Wallis test) or consumers’ perceptions (*p* = 0.26, as per the generalized linear model), meaning that the nutritional quality of the soups did not influence participants’ ability to better recall or influence their perceptions. Therefore, participants were combined into four groups based on the label they were shown: (1) No Claim No Symbol, (2) Nutrient Content Claim, (3) Symbol depicting ‘health,’ and (4) Claim + Symbol (Figure 1). 

### 3.1. Claim Recall and Understanding of the Claim

The results showed that most participants who saw the soup with the claim displayed on the label were able to recall it (Table 2), with 73% (n = 340/463) of participants in group 2 and 76% of participants in group 4 (n = 352/463) correctly recalling the claim. We also found that 12% (n = 55/457) and 11% (n = 50/455) of participants in groups 1 and 3, respectively, thought they had read a claim, even though no claim was displayed on those labels. Of the 1997 participants, only 33% (n = 658) provided a description of the claim, and 30% (n = 595) of participants provided a statement about their understanding of the claim, mostly given by those in groups 2 and 4 (Table 2). When responses were analyzed from those who provided feedback, we found that most participants identified the claim as being related to fats (68%). Interestingly, 16% mentioned a “sodium-related” claim alongside a fat claim, although no mention of sodium was made on package. Likewise, when asked about the meaning of the claim, 50% of participants interpreted the claim as being related to fat, 27% thought the product was healthy, and 11% of participants thought the product had a sodium-related claim in addition to fat claims.

### 3.2. Perceived Nutritional Quality and Purchase Intentions

**Figure 3:**
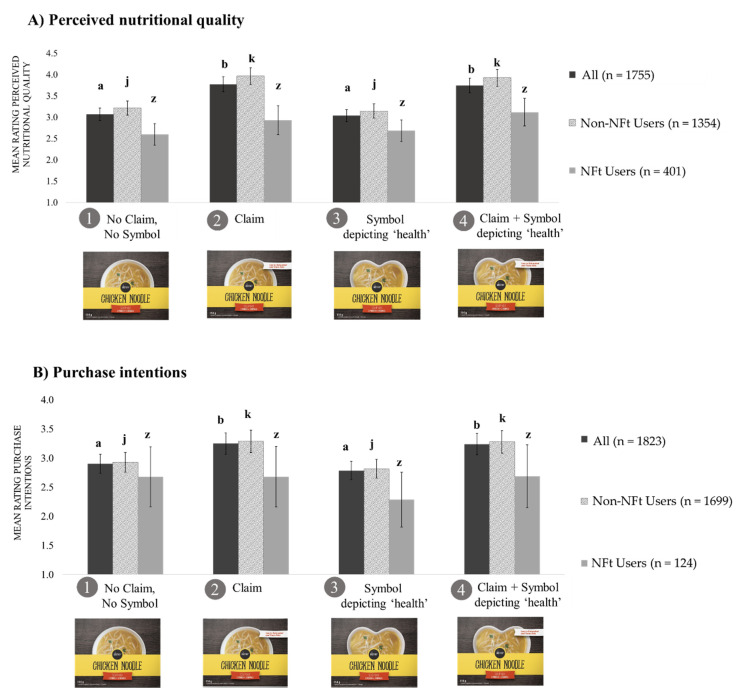
Means ratings of perceived nutritional quality (**A**) and purchase intentions (**B**) by condition overall and by Nutrition Facts table use (n = 1997)^1,2,3^. ^1^ Adjusted for gender, education, income, ethnicity, and health literacy. ^2^ Error bars show the standard error of the mean (SEM). ^3^ Because the nutritional quality of soups had no effect, participants were combined into four groups based on the label they were shown: (1) No Claim No Symbol, (2) Nutrient Content Claim, (3) Symbol depicting ‘health,’ and (4) Nutrient Content Claim + Symbol depicting ‘health.’ ^a,b^ Dark gray bars (All Participants), (means ± SEM ratings by all participants) with different superscript letters indicated statistically significant differences at *p* < 0.001 by the generalized linear model with Bonferroni correction. ^j,k^ Light grey bars (Non-NFt users), (means ± SEM ratings by Non-NFt users) with different superscript letters indicated statistically significant differences at *p* < 0.001 by the generalized linear model with Bonferroni correction. ^z^ Medium grey bars (NFt users), (means ± SEM ratings by NFt users) with different superscript letters indicated statistically significant differences at *p* < 0.001 by the generalized linear model with Bonferroni correction. Separate generalized linear models were conducted for each of the three.

Figure 3 shows consumers perceptions (details in Supplementary Table S2). As expected, products displaying the claim (Figure 3A, “All,” groups 2, 4) were perceived as healthier than those without (i.e., ‘halo’ effect). However, the health-related symbol did not magnify the effect of a claim as hypothesized, with no significant differences between participants in groups 2 and 4 (pairwise comparison *p* = 1). 

Only 23% (n = 401/1755) of participants clicked on the NFt link (Figure 3A). Among participants who did not click the NFt link (“Non-NFt users”), soups with a claim were perceived as healthier compared to those without the claim (*p* < 0.001). For “NFt users” (Figure 3A), we did not find significant differences. Whereas we did not find significant differences between healthier and less healthy soups among all participants, we found that NFt users were able to discriminate products with varying nutritional composition. For example, NFt users perceived the soup with better nutritional quality as healthier (mean = 3.2, Confidence Intervals (CI) = 2.9–3.4) compared to the less healthy soup (mean = 2.5, CI = 2.3–2.7, *p* < 0.001). 

Regarding purchase intentions, we found similar results on perceived nutritional quality (Figure 3B). Participants had significantly higher purchase intentions for soups with a claim (group 2, mean = 3.3, CI = 3.0–3.5) and soups with a claim and symbol (group 4, mean = 3.2, CI = 3.0–3.5) than for soups without a claim (group 1, mean = 2.9, CI = 2.7–3.1) and soups with a symbol only (group 3, mean = 2.6, CI = 2.6–3.0). Whereas only 7% (n = 124/1823) of participants clicked on the NFt link when asked about their purchase intentions, we did not find any significant differences in purchase intentions between groups (*p* = 0.61). Detailed information is provided in Supplementary Table S2.

We did not collect information on the time that participants may have looked at/evaluated the NFt.

### 3.3. Sensitivity Analyses

An interesting finding was that male participants tended to rate products “healthier” than female participants. However, overall, both genders perceived products with claims as “healthier” than products without claims (Table 3). A similar result was found when products were assessed by level of health literacy. Participants with likely/possible low level of health literacy rated products as “healthier” compared to those with adequate health literacy (Table 3). Yet, our main finding was consistent with what we have identified: Products with claims were perceived as “healthier” than those without claims.

## 4. Discussion

This study assessed consumers’ recall and understanding of nutrition claims and health-related symbols (through an assessment of their implicit evaluation of labels) and their impact on perceived nutritional quality and purchase intentions (through an assessment of their implicit/explicit evaluation of labels). 

Overall, our results support earlier observations that nutrition claims likely attract consumers’ attention [29]. For instance, more than 70% of participants that had a claim displayed on the label (groups 2 and 4) were able to recall it, despite labels being shown for only 10 s. Our results support previous research that has identified, using eye-tracking technology, that nutrition information is one of the most viewed elements on a food label [13,18,30]. 

Importantly, we also found that 16% of participants mentioned a sodium-related message (in conjunction with fat claims), despite no claim or symbol showing that information. This finding could be related to participants’ preconceptions about levels of sodium in soups. Perhaps for some participants, product assessment was based on recall of information ‘previously learned’ (i.e., through System 1 [1]), which could have triggered their memory to associate that soups often carry sodium claims, as shown in previous Canadian research [60]. 

This study also showed that nutrition claims were more likely to create a ‘halo’ effect, regardless of product’s nutritional quality, especially among the majority who did not click on the NFt. This finding is in line with a growing body of evidence [47,49,62,68,69,70,71,72,73], suggesting that products with nutrition claims are perceived “healthier,” and therefore, nutrition claims are more likely to be used as a marketing tool rather than to promote healthier food choices among consumers. These results are worrying, as claims are often used on ultra-processed foods [38,74,75]. We also found that, without an accompanying written claim, an image implying a health function [58] (i.e., heart-shaped bowl) did not enhance the ‘halo’ effect already created by the claim, as we did not find significant differences on the ratings of perceived nutritional quality between products with a claim and products with both a claim and symbol, even among Non-NFt users. Similar results were also found when assessing purchase intentions. 

Results demonstrated that consumers were more likely to assess products quickly using the information provided on the front of the label (likely using System 1, fast decision-making), as only one-quarter of participants in this sample clicked on the NFt link. This proportion, which is much lower than what has been self-reported [76], is probably a more accurate reflection of the actual purchase situation at the point of sale. Importantly, previous research has shown that attention to labels can be significantly disrupted when there is a time constraint [18]. It could be hypothesized that, because the information presented on the NFt requires time and a cognitive process by consumers (i.e., requires consumers to transition from System 1 to System 2 thinking), the NFt therefore is less preferable than short-text claims and images that are already “interpreting” nutrition information for consumers. This assumption concurs with published psychology research concluding that consumers more often rely on System 1 than on System 2 to make choices [1]. 

As consumers may not often use the NFt or have enough time to thoroughly assess product labels when shopping, FOP labelling that displays nutrition information in a prominent, easy, and simple way [77,78,79] might be ideally suited for “System 1 fast decision-making.” FOP labelling can attract consumers’ attention more quickly compared to nutrition claims [79]. FOP labelling, which highlights nutrients of public health concern, could be very useful for consumers, particularly when trying to quickly differentiate between healthier and less healthy food options, and might counterbalance the ‘halo’ effect created by claims, especially for less healthy foods [49,79]. 

As an online survey, this study might not represent in-store consumer behaviors. However, as technology is expanding toward a more digital world, the prevalence of online grocery shopping will likely increase. It is important to note that this study was conducted in 2017, more than two years prior the global pandemic of COVID-19. Thus, many of our participants may not have experienced online grocery shopping before engaging in this study. Given the growth of online grocery shopping in the current environment, it might be worth repeating this study to determine whether the same results are found. Another limitation is that participants were required to have a smartphone, and therefore, the sample is not representative of the population in Canada. However, in 2017, over 90% of the population had access to Long-Term Evolution Advance network services [80]. We only evaluated one type of food (soup), and therefore, extrapolation to other foods and beverages is limited. Another limitation is that the ‘heart-shaped’ symbol depicting ‘health’ may not have been visible enough to attract consumers’ attention. We also were not able to identify what the first element of the label viewed by participants was or how long they observed a certain element or characteristic. Eye-tracking technology can overcome this shortcoming [18,30,79]. This study was strengthened by the use of a randomized experimental design and a large sample size calculated to have statistical power to detect differences among all groups, and by including healthier and less healthy versions of the same product.

## 5. Conclusions

This study raised important issues about the use of nutrition claims on food labels. As discussed, claims likely attract consumers’ attention. However, not all consumers may identify or understand the message of claims correctly. Thus, claims can mislead consumers. The presence of the claim increased perceived nutritional quality (i.e., the ‘halo’ effect) on consumers, regardless of the product’s nutritional composition, which is particularly concerning given the number of less healthy products that might display nutrition claims on their labels. Those few participants who clicked on and likely used the NFt had a better understanding of the nutritional quality of the product. Therefore, the influence of the claim was reduced. The influence of the health-related heart-shaped symbol was limited. As consumers are less likely to use nutrient declarations when assessing and/or purchasing foods, results from this study stress the importance of providing consumers with nutrition labels that *also* support fast decision-making.

## Figures and Tables

**Table 1:** Participants’ characteristics (n = 1997).

Demographics		Clicked at the NFt when Assessing Nutritional Quality		Clicked at the NFt when Assessing Purchase Intentions	
	All	Non-NFt Users	NFt Users	Non-NFt Users	NFt Users
	(n = 1997)	(n = 1554)	(n = 443)	(n = 1860)	(n = 137)
**Age (years)**	**n (%)**	**n (%)**	**n (%)**	**n (%)**	**n (%)**
18-25	256 (12.8)	225 (14.5)	43 (9.7)	242 (13.0)	14 (10.2)
26-35	652 (32.6)	528 (34.0)	112 (25.3)	621 (33.4)	31 (22.6)
36-45	493 (24.7)	384 (24.7)	109 (24.6)	459 (24.7)	34 (24.8)
46-55	359 (18.0)	251 (16.2)	108 (24.4)	328 (17.6)	31 (22.6)
56-65	176 (8.8)	121 (7.8)	55 (12.4)	157 (8.4)	19 (13.9)
66+	61 (3.1)	45 (2.9)	16 (3.6)	53 (2.8)	8 (5.8)
Refused	0 (0.0)	0 (0.0)	0 (0.0)	0 (0.0)	0 (0.0)
**Gender**	**n (%)**	**n (%)**	**n (%)**	**n (%)**	**n (%)**
Male	957 (47.9)	764 (49.2)	193 (43.6)	892 (48.0)	65 (47.4)
Female	1037 (51.9)	789 (50.8)	248 (56.0)	965 (51.9)	72 (52.6)
Another	3 (0.2)	1 (0.1)	2 (0.5)	3 (0.2)	0 (0.0)
**Education**	**n (%)**	**n (%)**	**n (%)**	**n (%)**	**n (%)**
Did not graduate high school	36 (1.8)	27 (1.7)	9 (2.0)	32 (1.7)	4 (2.9)
High school certificate or equivalent	324 (16.2)	255 (16.4)	69 (15.6)	305 (16.4)	19 (13.9)
Trades certificate or diploma	99 (4.9)	73 (4.7)	26 (5.9)	91 (4.9)	8 (5.8)
Community college, technical college, or CEGEP	511 (25.6)	382 (24.6)	129 (29.1)	475 (25.5)	36 (26.3)
University (undergraduate degree)	762 (38.2)	617 (39.7)	145 (32.7)	715 (38.4)	47 (34.3)
Post-graduate degree (Masters, PhD)	259 (13)	194 (12.5)	65 (14.7)	236 (12.7)	23 (16.8)
Not stated	6 (0.3)	6 (0.4)	0 (0.0)	6 (0.3)	0 (0.0)
**Ethnicity**	**n (%)**	**n (%)**	**n (%)**	**n (%)**	**n (%)**
White	1375 (68.9)	1038 (66.8)	337 (76.1)	1264 (68.0)	111 (81.0)
Nonwhite	589 (29.5)	487 (31.3)	102 (23.0)	566 (30.4)	23 (16.8)
Not stated	33 (1.7)	29 (1.9)	4 (0.9)	30 (1.6)	3 (2.2)
**Household income**	**n (%)**	**n (%)**	**n (%)**	**n (%)**	**n (%)**
$25,000 or less	169 (8.5)	134 (8.6)	35 (7.9)	164 (8.8)	5 (3.6)
$25,000–$49,999	373 (18.7)	290 (18.7)	83 (18.7)	356 (19.1)	17 (12.4)
$50,000–$74,999	409 (20.5)	322 (20.7)	87 (19.6)	384 (20.6)	25 (18.2)
$75,000–$99,999	338 (16.9)	269 (17.3)	69 (15.6)	304 (16.3)	34 (24.8)
$100,000–$124,999	274 (13.7)	218 (14.0)	56 (12.6)	253 (13.6)	21 (15.3)
$125,000 or more	288 (14.4)	213 (13.7)	75 (16.9)	265 (14.2)	23 (16.8)
Not stated	146 (7.3)	108 (6.9)	38 (8.6)	134 (7.2)	12 (8.8)
**Language primarily spoken at home**	**n (%)**	**n (%)**	**n (%)**	**n (%)**	**n (%)**
English	1830 (91.6)	1427 (91.8)	403 (91.0)	1701 (91.5)	129 (94.2)
French	44 (2.2)	29 (1.9)	15 (3.4)	41 (2.2)	3 (2.2)
Other	118 (5.9)	93 (6.0)	25 (5.6)	113 (6.1)	5 (3.6)
Not stated	5 (0.3)	5 (0.3)	0 (0.0)	5 (0.3)	0 (0.0)
**Dependent children (<18 years)**	**n (%)**	**n (%)**	**n (%)**	**n (%)**	**n (%)**
Yes	758 (38)	591 (38.0)	167 (37.7)	710 (38.2)	48 (35.0)
No	1229 (61.5)	954 (61.4)	275 (62.1)	1140 (61.3)	89 (65.0)
Not stated	10 (0.5)	9 (0.6)	1 (0.2)	10 (0.5)	0 (0.0)
Health literacy *	**n (%)**	**n (%)**	**n (%)**	**n (%)**	**n (%)**
Likely low health literacy	202 (10.1)	197 (12.7)	5 (1.1)	200 (10.8)	2 (1.5)
Possible low health literacy	263 (13.2)	226 (14.5)	37 (8.4)	251 (13.5)	12 (8.8)
Adequate health literacy	1528 (76.5)	1127 (72.5)	401 (90.5)	1405 (75.5)	123 (98.8)
Missing	4 (0.2)	4 (0.3)	0 (0.0)	4 (0.2)	0 (0.0)

* Assessed with the Newest Vital Sign questionnaire [64,65].

**Table 2:** Responses to open-ended questions assessing consumers’ recall and understanding of the claim ^1,2^.

	All		(1) No Claim No Symbol ^2^		(2) Claim ^2^		(3) Symbol ^2^		(4) Claim + Symbol ^2^	
	n = 1997		n = 496		n = 500		n = 503		n = 498	
	*n*	%responses	*n*	%responses	*n*	%responses	*n*	%responses	*n*	% responses
**In response to *“To the best of your recollection, was there a claim regarding the nutritional quality of the product that you were shown?”***										
Yes	797	43.4%	55	12.0%	340	73.4%	50	11.0%	352	76.0%
No	1041	56.6%	402	88.0%	123	26.6%	405	89.0%	111	24.0%
**Total responses**	**1838**	**100%**	**457**	**100%**	**463**	**100%**	**455**	**100%**	**455**	**100%**
**In response to *“What did the claim describe?”***										
Low sat fat and trans fat, low fat	449	68.2%	1	4.5%	210	71.7%	0	0.0%	238	73.2%
Low in sodium, fat, sat fat, trans fat	104	15.8%	1	4.5%	53	18.1%	2	11.1%	48	14.8%
Healthy, hearty, OK, good for you	33	5.0%	3	13.6%	13	4.4%	6	33.3%	11	3.4%
Number of calories, Net Weight, servings	24	3.6%	10	45.5%	2	0.7%	3	16.7%	9	2.8%
Chicken noodle soup, great soup, convenience, reliability, taste, quality	18	2.7%	6	27.3%	4	1.4%	4	22.2%	4	1.2%
Low calories, cholesterol, sugars, any fat	17	2.6%	0	0.0%	9	3.1%	0	0.0%	8	2.5%
Can’t remember, not sure	10	1.5%	0	0.0%	1	0.3%	2	11.1%	7	2.2%
Not healthy, high in sodium	3	0.5%	1	4.5%	1	0.3%	1	5.6%	0	0.0%
**Total responses**	**658**	**100.0%**	**22**	**100.0%**	**293**	**100.0%**	**18**	**100.0%**	**325**	**100.0%**
**In response to “*What do you think this claim means?”***										
Low fat (sat, trans, total)	296	49.7%	0	0.0%	144	53.9%	0	0.0%	152	52.8%
Healthy, healthier choice, better choice, good for you, nutrition value, OK	159	26.7%	13	54.2%	64	24.0%	9	56.3%	73	25.3%
Low in salt/sodium, low in fats (sat, trans, total)	66	11.1%	0	0.0%	36	13.5%	3	18.8%	27	9.4%
Nothing, not much, not sure, neutral, marketing	36	6.1%	4	16.7%	11	4.1%	2	12.5%	19	6.6%
Less calories, sugars, cholesterol, low in any fat	16	2.7%	0	0.0%	4	1.5%	0	0.0%	12	4.2%
Homestyle, homemade, minimal processed, tasty, great soup, noodle soup	9	1.5%	3	12.5%	3	1.1%	1	6.3%	2	0.7%
Number of calories, Nt Wt, servings, portions	7	1.2%	4	16.7%	1	0.4%	0	0.0%	2	0.7%
Contains ± chemicals, flavors, fiber	6	1.0%	0	0.0%	4	1.5%	1	6.3%	1	0.3%
**Total responses**	**595**	**100.0%**	**24**	**100.0%**	**267**	**100.0%**	**16**	**100.0%**	**288**	**100.0%**

^1^ The open-ended responses from participants contained multiple but similar key themes. ^2^ Because the nutritional quality did not have significant effects in either consumers’ recall or consumers’ perceptions, therefore participants were combined into four groups based on the label they were shown: (1) No Claim No Symbol, (2) Nutrient Content Claim, (3) Symbol depicting ‘health,’ and (4) Nutrient Content Claim + Symbol depicting ‘health’.

**Table 3:** Means ratings of perceived nutritional quality and purchase intentions by (a) gender and (b) health literacy (n = 1997) ^1^.

Perceived Nutritional Quality							
(a) Gender ^2^		Males (n = 843)			Females (n = 909)		
		Mean	95% CI	*p*-value ^4^	Mean	95% CI	*p*-value ^4^
Group 1	No Claim, No Symbol	3.2	3.0–3.5	*p* < 0.001	2.9	2.7–3.2	*p* < 0.001
Group 2	Claim	3.9	3.6–4.3		3.6	3.3–4.0	
Group 3	Symbol	3.1	2.9–3.4		3.0	2.7–3.3	
Group 4	Claim + Symbol	3.9	3.6–4.3		3.6	3.3–3.9	
**(b) Health literacy ^3^**		**Likely/possible low health literacy (n = 416)**			**Adequate health literacy (n = 1339)**		
		Mean	95% CI	*p*-value ^4^	Mean	95% CI	*p*-value ^4^
Group 1	No Claim, No Symbol	4.1	3.6–4.7	*p* = 0.001	2.8	2.6–3.0	*p* < 0.001
Group 2	Claim	4.5	4.0–5.0		3.6	3.3–3.8	
Group 3	Symbol	3.5	3.1–4.0		2.9	2.7–3.1	
Group 4	Claim + Symbol	4.5	3.9–5.0		3.6	3.3–3.8	
Purchase Intentions							
**(a) Gender ^2^**		**Males (n = 890)**			**Females (n = 930)**		
		Mean	95% CI	*p*-value ^4^	Mean	95% CI	*p*-value ^4^
Group 1	No Claim, No Symbol	3.0	2.7–3.3	*p* = 0.001	2.8	2.5–3.1	*p* = 0.076
Group 2	Claim	3.4	3.1–3.8		3.1	2.8–3.4	
Group 3	Symbol	2.9	2.6–3.2		2.7	2.5–3.0	
Group 4	Claim + Symbol	3.4	3.1–3.8		3.1	2.8–3.4	
**(b) Health literacy ^3^**		**Likely/possible low health literacy (n = 424)**			**Adequate health literacy (n = 1399)**		
		Mean	95% CI	*p*-value ^4^	Mean	95% CI	*p*-value ^4^
Group 1	No Claim, No Symbol	3.9	3.4–4.5	*p* = 0.012	2.6	2.4–2.9	*p* = 0.001
Group 2	Claim	4.4	3.8–5.0		2.9	2.7–3.1	
Group 3	Symbol	3.4	3.0–3.9		2.6	2.4–2.8	
Group 4	Claim + Symbol	4.1	3.5–4.7		3.0	2.8–3.3	

^1^ Because the nutritional quality of soups had no effect, participants were combined into four groups based on the label they were shown: (1) No Claim No Symbol, (2) Nutrient Content Claim, (3) Symbol depicting ‘health,’ and (4) Nutrient Content Claim + Symbol depicting ‘health.’^2^ Adjusted for education, income, ethnicity, and health literacy. ^3^ Adjusted for education, income, ethnicity, and gender. ^4^ As determined by generalized linear models. CI—Confidence Intervals.

## Supplementary Materials

The following are available online at https://www.mdpi.com/1660-4601/17/21/8213/s1, Figure S1: CONSORT Diagram, Table S1: CONSORT checklist of information to include when reporting a randomized trial, Table S2. Means ratings of perceived nutritional quality (**a**) and purchase intentions (**b**) by condition overall and by Nutrition Facts table use.
