# Supplementary material for: Consumers’ Implicit and Explicit Recall, Understanding and Perceptions of Products with Nutrition-Related Messages: An Online Survey
Source: Int J Environ Res Public Health. 2020 Nov 6;17(21):8213. doi: 10.3390/ijerph17218213 (PMC7664426; doi:10.3390/ijerph17218213)
Supplement: Supplementary file 1 [file ijerph-17-08213-s001.pdf]

Supplementary Materials

Figure S1. CONSORT Diagram

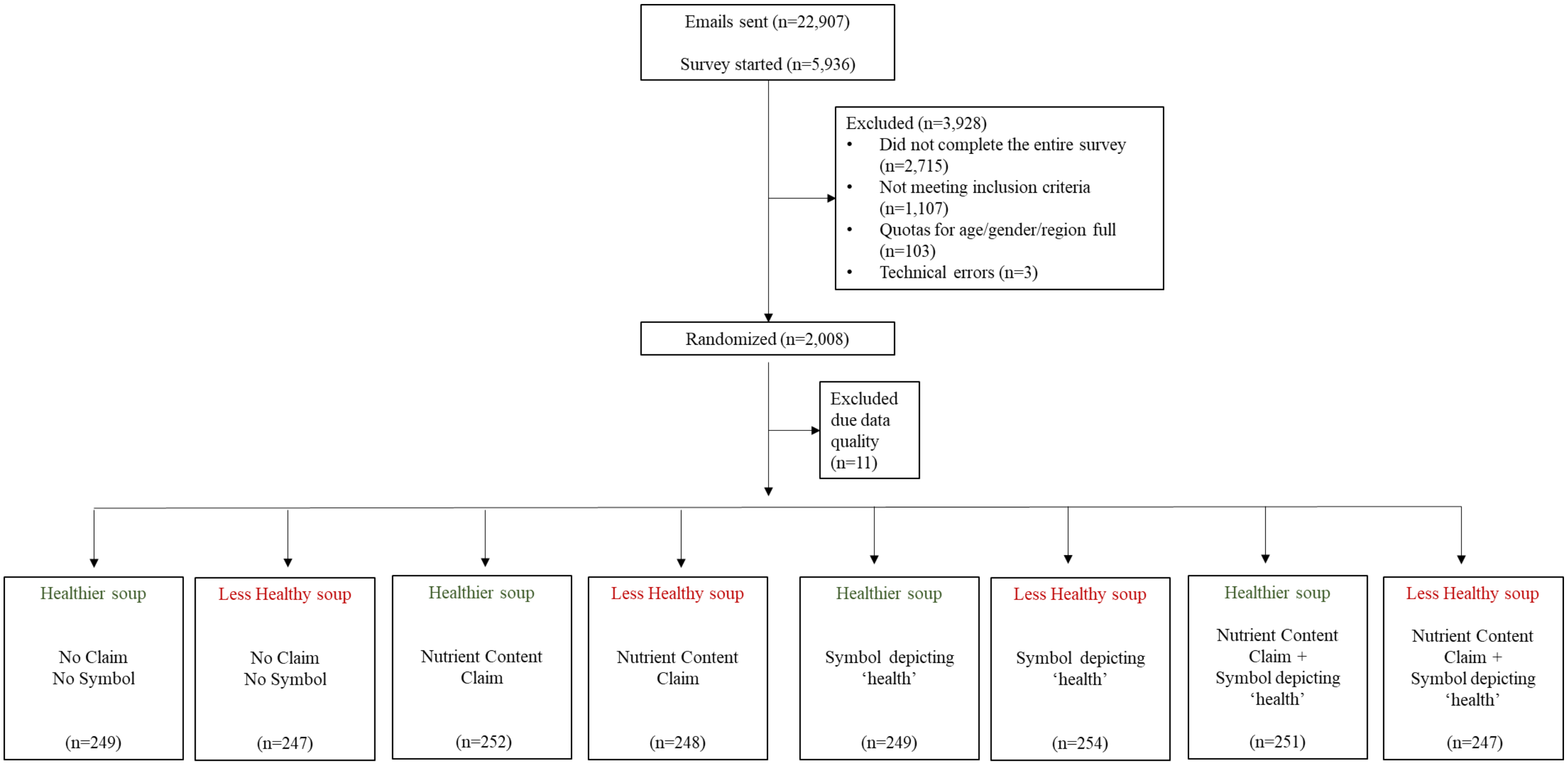

Table S1. CONSORT checklist of information to include when reporting a randomized trial

Section/Topic	Item No	Checklist item	Reported on page No
Title and abstract			
	1a	Identification as a randomized trial in the title	n/a
	1b	Structured summary of trial design, methods, results, and conclusions (for specific guidance see CONSORT for abstracts)	Subjects/ Methods
Introduction			
Background and objectives	2a	Scientific background and explanation of rationale	Introduction: paragraphs 1–4
	2b	Specific objectives or hypotheses	Introduction: paragraph 6
Methods			
Trial design	3a	Description of trial design (such as parallel, factorial) including allocation ratio	Methods: *Experimental design & stimuli* paragraph 1
	3b	Important changes to methods after trial commencement (such as eligibility criteria), with reasons	Methods: *Survey design* paragraph 2
Participants	4a	Eligibility criteria for participants	Methods: *Survey design* paragraph 1
	4b	Settings and locations where the data were collected	Methods: *Survey design* paragraph 1
Interventions	5	The interventions for each group with sufficient details to allow replication, including how and when they were actually administered	Methods: *Experimental design & stimuli* paragraphs 1-4
Outcomes	6a	Completely defined pre-specified primary and secondary outcome measures, including how and when they were assessed	Methods: *Analyses* paragraph 1
	6b	Any changes to trial outcomes after the trial commenced, with reasons	
Sample size	7a	How sample size was determined	Methods: *Experimental design & stimuli* paragraph 1
	7b	When applicable, explanation of any interim analyses and stopping guidelines	
Randomisation:			
Sequence generation	8a	Method used to generate the random allocation sequence	Methods: *Experimental design & stimuli* paragraph 1
	8b	Type of randomisation; details of any restriction (such as blocking and block size)	Methods: *Experimental design & stimuli* paragraph 1
Allocation concealment mechanism	9	Mechanism used to implement the random allocation sequence (such as sequentially numbered containers), describing any steps taken to conceal the sequence until interventions were assigned	Methods: *Experimental design & stimuli* paragraph 1
Implementation	10	Who generated the random allocation sequence, who enrolled participants, and who assigned participants to interventions	Methods: *Survey design* paragraph 1

Blinding	11a	If done, who was blinded after assignment to interventions (for example, participants, care providers, those assessing outcomes) and how	
	11b	If relevant, description of the similarity of interventions	
Statistical methods	12a	Statistical methods used to compare groups for primary and secondary outcomes	Methods: *Analyses* paragraph 1
	12b	Methods for additional analyses, such as subgroup analyses and adjusted analyses	Methods: *Analyses* paragraph 1
Results			
Participant flow (a diagram is strongly recommended)	13a	For each group, the numbers of participants who were randomly assigned, received intended treatment, and were analysed for the primary outcome	Supplementary material: CONSORT figure 1
	13b	For each group, losses and exclusions after randomisation, together with reasons	Supplementary material: CONSORT figure 1
Recruitment	14a	Dates defining the periods of recruitment and follow-up	Methods: *Survey design* paragraph 1
	14b	Why the trial ended or was stopped	
Baseline data	15	A table showing baseline demographic and clinical characteristics for each group	Table 1
Numbers analysed	16	For each group, number of participants (denominator) included in each analysis and whether the analysis was by original assigned groups	Fig 3
Outcomes and estimation	17a	For each primary and secondary outcome, results for each group, and the estimated effect size and its precision (such as 95% confidence interval)	Table 3, Supplementary material: Supplementary Table 2
	17b	For binary outcomes, presentation of both absolute and relative effect sizes is recommended	
Ancillary analyses	18	Results of any other analyses performed, including subgroup analyses and adjusted analyses, distinguishing pre-specified from exploratory	
Harms	19	All important harms or unintended effects in each group (for specific guidance see CONSORT for harms)	
Discussion			
Limitations	20	Trial limitations, addressing sources of potential bias, imprecision, and, if relevant, multiplicity of analyses	Discussion: paragraph 7
Generalisability	21	Generalizability (external validity, applicability) of the trial findings	Discussion: paragraph 7
Interpretation	22	Interpretation consistent with results, balancing benefits and harms, and considering other relevant evidence	
Other information			
Registration	23	Registration number and name of trial registry	Methods: *Survey design* paragraph 1
Protocol	24	Where the full trial protocol can be accessed, if available	Methods: *Survey design* paragraph 1
Funding	25	Sources of funding and other support (such as supply of drugs), role of funders	Funding and Conflict of interest paragraphs

Table S2. Means ratings of perceived nutritional quality (a) and purchase intentions (b) by condition overall and by Nutrition Facts table use (n=1,997)[1,2,3]

**Perceived nutritional quality**	**All (n=1,755)**		**Non-NFt Users (n=1,634)**		**NFt Users (n=401)**	
	Mean	95% CI	Mean	95% CI	Mean	95% CI
No Claim, No Symbol	3.1[a]	2.9-3.2	3.2[j]	3.0-3.5	2.6[z]	2.3-3.0
Claim	3.8[b]	3.6-4.0	4.0[k]	3.7-4.2	2.9[z]	2.5-3.4
Symbol	3.0[a]	2.9-3.2	3.2[j]	3.0-3.4	2.7[z]	2.4-3.0
Claim+Symbol	3.8[b]	3.6-3.9	3.9[k]	3.7-4.2	3.1[z]	2.7-3.6

**Purchase intentions**	**All (n=1,823)**		**Non-NFt Users (n=1,699)**		**NFt Users (n=124)**	
	Mean	95% CI	Mean	95% CI	Mean	95% CI
No Claim, No Symbol	2.9[a]	2.7-3.1	2.9[j]	2.7-3.2	2.7[z]	2.1-3.5
Claim	3.3[a]	3.0-3.5	3.3[k]	3.0-3.6	2.7[z]	2.1-3.5
Symbol	2.8[a]	2.6-3.0	2.8[j]	2.6-3.0	2.3[z]	1.8-3.0
Claim+Symbol	3.2[a]	3.0-3.5	3.3[k]	3.0-3.5	2.7[z]	2.1-3.5

[1]Adjusted for gender, education, income, ethnicity and health literacy. [2]Means with different superscripts were significantly different (Bonferroni corrections for multiple comparisons, p<0.001). [3]Because the nutritional quality of soups had no effect on recalling the claim, participants were combined into four groups based on the label they were shown: 1) No Claim No Symbol, 2) Nutrient Content Claim, 3) Symbol depicting 'health' and 4) Nutrient Content Claim+Symbol depicting 'health'. CI – Confidence Intervals; NFt – Nutrition Facts table
